# Dr. Mordaunt Stewart Robinson

**Published:** 1847-10

**Authors:** 


*Obituary.*—Died in Valparaiso de Chili, on the 14th of July last, of inflammation of the liver, Dr. Mordaunt Stewart Robinson, youngest son of N. N. Robinson, Esq. of Baltimore. He was a graduate of the Baltimore College of Dental Surgery, and at his examination received the highest testimonials which it was in the power of his preceptor to award him. He left Baltimore on 2nd of June, 1845, for Valparaiso, to practice his profession in connection with his brother Dr. B. R. Robinson, who had taken up his residence there.

He was destined to be one of the brightest ornaments to his profession, for, combined with his courteous and gentlemanly bearing, he was in every respect qualified to administer to those who should require his services in each and every branch of his business.

The writer of this imperfect sketch enjoyed a long and uninterrupted intimacy with him, and sincerely sympathizes with his family and friends in their sore affliction, but they have abundant consolation in knowing that their loss is his gain, for he was not unmindful of his spiritual welfare, but expressed himself to be perfectly resigned to his fate and subservient to the will of his God. "Blessed are the dead who die in the Lord."

R. W. T.—D. D. S.

